# Carbon Nano-Onions Reinforced Multilayered Thin Film System for Stimuli-Responsive Drug Release

**DOI:** 10.3390/pharmaceutics12121208

**Published:** 2020-12-13

**Authors:** Narsimha Mamidi, Ramiro Manuel Velasco Delgadillo, Aldo Gonzáles Ortiz, Enrique V. Barrera

**Affiliations:** 1 Department of Chemistry and Nanotechnology, School of Engineering and Sciences, Tecnologico de Monterrey, Ave. Eugenio Garza Sada 2501, Monterrey NL-64849, Mexico; a01233475@itesm.mx (R.M.V.D.); aldogortiz@gmail.com (A.G.O.); 2 Department of Materials Science and Nanoengineering, Rice University, Houston, TX 77005, USA; ebarrera@rice.edu

**Keywords:** nanocomposite thin films, layer-by-layer assembly, strength, Young’s modulus, toughness, pH-responsive drug release, cell viability

## Abstract

Herein, poly (*N*-(4-aminophenyl) methacrylamide))-carbon nano-onions (PAPMA-CNOs = f-CNOs) and anilinated-poly (ether ether ketone) (AN-PEEK) have synthesized, and AN-PEEK/f-CNOs composite thin films were primed via layer-by-layer (LbL) self-assembly for stimuli-responsive drug release. The obtained thin films exhibited pH-responsive drug release in a controlled manner; pH 4.5 = 99.2% and pH 6.5 = 59.3% of doxorubicin (DOX) release was observed over 15 days. Supramolecular π–π stacking interactions between f-CNOs and DOX played a critical role in controlling drug release from thin films. Cell viability was studied with human osteoblast cells and augmented viability was perceived. Moreover, the thin films presented 891.4 ± 8.2 MPa of the tensile strength ($\sigma_{ult}$), 43.2 ± 1.1 GPa of Young’s modulus (E), and 164.5 ± 1.7 Jg^−1^ of toughness (K). Quantitative scrutiny revealed that the well-ordered aligned nanofibers provide critical interphase, and this could be responsible for augmented tensile properties. Nonetheless, a pH-responsive and mechanically robust biocompatible thin-film system may show potential applications in the biomedical field.

## 1. Introduction

In the past few decades, nanocarriers-based drug delivery systems (NDDSs) have received significant attention in cutting-edge nanomedicine due to their amended pharmacokinetics and pharmacodynamics [1,2]. Owing to impressive growth in pharmaceutics, and materials science, a broad range of NDDSs have developed. For instance, liposomes, micelles, dendrimers, nanoparticles, nanofibers, hydrogels, and thin films [3,4,5,6,7]. Among them, stimuli-responsive thin films have been used as potential NDDS with promising applications in drug delivery [6,8]. Recently, the stimuli-responsive strategies have become a key objective in nanomedicine, especially for chemotherapeutic drug delivery [3,5,9]. The stimuli strategies can be categorized into two classes such as intrinsic and extrinsic stimuli-responsive systems. Exogenous stimuli include temperature, light, ultrasounds, magnetic fields, and electric fields, whereas intrinsic stimuli include changes in pH, protein/enzyme levels, redox potential, and others of the tumor microenvironments. These stimuli cause a significant variation in the physicochemical properties or chemical structure of nanocarriers.

However, the development of efficient thin films desires a suitable manufacturing method along with comprehensive knowledge of the pharmaceutical and pharmacological properties of polymers and drugs. Therefore, various methods including self-assembled monolayer techniques, Langmuir–Blodgett method, layer-by-layer (LbL) self-assembly and magnetic fields have been utilized to fabricate thin films [10,11,12,13,14,15]. Among these strategies, LbL self-assembly is one of the efficient processes to fabricate stimuli-responsive thin films with specific interior nanostructures, controlled material composition, and physicochemical properties [16,17]. This process allows the growth of multilayer thin films with a “sandwich” like internal architecture, and the capability to carry a wide range of chemotherapeutic reagents. LbL self-assembly of thin films has compelled by physical molecular interactions without chemical precursors, which offers a highly nontoxic and biocompatible system. Moreover, within the thin films, the structures, quantity, and proportions of the multilayer components can be manipulated at the nanoscale level. The characteristics of LbL thin films rely on the intrinsic physicochemical properties of the building blocks. Therefore, it is crucial to select suitable nanomaterials to prepare building blocks.

A wide variety of materials such as micelles, nanoparticles, polyelectrolytes, and proteins has been used as building blocks for LbL-self-assembly thin films [18,19,20,21]. In this process, thin films are formed through the step-by-step deposition of monolayers of individual materials via hydrogen bonds, van der Waals, electrostatic interactions, and bio-specific interactions. These features allow the controlled release of the drug from the thin films incorporated with therapeutic agents. Therefore, there is a clear unmet need to design novel thin-film systems with superior physicochemical properties. Carbon nano-onions (CNOs) are the new class of emerging carbon fillers that display great potential in many applications. CNOs structures persist as one of the most exciting and fascinating carbon forms, along with graphene and its derivatives [22,23]. Their exceptional biocompatibility and biosafety make them promising nanofillers for cell imaging and a variety of biomedical applications [24,25,26]. The CNOs exhibit a high specific surface area that realizes in the establishment of a large structural interface, hence strong interactions with the polymer matrix. In this regard, poly mercaptophenyl methacrylated (PMPMA) and 4-hydroxyphenyl methacrylated (PHPMA) CNOs have synthesized, and incorporated within zein, PCL, gelatin, and bovine serum albumin (BSA) protein matrixes. The resulting nanocomposites exhibited amended stimuli drug release, cytocompatibility, thermal and mechanical properties [27,28,29,30].

Thus, our previous studies have revealed that stimuli-responsive f-CNOs demonstrated high drug loading efficiency and a sustained controlled drug release rate, which makes them be an effective drug delivery system. Motivated by these exceptional advantages, we have designed and synthesized poly (*N*-(4-aminophenyl) methacrylamide))-carbon nano-onions (PAPMA-CNOs = f-CNOs) as an effective nanofiller system to fabricate anilinated-poly (ether ether ketone) (AN-PEEK)/f-CNOs thin films. PEEK is a thermoplastic polymer, and it has been used to replace metal implant components in orthopedics and trauma, owing to its excellent biocompatibility and mechanical properties comparable to human bones [31]. Besides, PEEK is a well-known component of aerospace, electronics, automotive, consumer products, and widely used in nanocomposite materials [32]. It would be very attractive to use functionalized PEEK as a polymeric matrix in the nanocomposites due to its unique combinations of strength, stiffness, thermo-oxidative stability, and chemical resistance. However, the use of anilinated-PEEK as a polymer matrix in the fabrication of carbon nano-onions (CNOs) nanocomposite thin films by the LbL strategy has never been conducted. Therefore, we have synthesized AN-PEEK and reinforced with f-CNOs to fabricate AN-PEEK/f-CNOs nanocomposite thin films by LbL self-assembly strategy.

In the current study, doxorubicin (DOX) loaded multilayered nanocomposite thin films were manufactured from AN-PEEK and f-CNOs fiber dispersions by the LbL strategy for the assessment of stimuli-responsive drug release. The physicochemical properties of composite thin films have studied systematically. The obtained thin films exhibited record-high tensile strength ($\sigma_{ult}$ = 891.4 ± 8.2 MPa), Young’s modulus (E = 43.2 ± 1.1 GPa), and toughness (K = 164.5 ± 1.7 Jg^−1^). The mechanical robust composite films can be twisted and bent. Benefitting from the f-CNOs incorporation, the thin films exhibited pH-responsive and sustained drug release. Moreover, improved cell viability was observed on the surface of thin films. The results demonstrated that AN-PEEK and f-CNOs are promising nanoscale building blocks with multiple fascinating characteristics.

## 2. Experimental Section

### 2.1. Materials

P-phenylenediamine, methacryloyl chloride, triethylamine (TEA), 2,2′-Azobis(2-methylpropionitrile) (AIBN), *N*-hydroxysuccinimide (NHS), 1-ethyl-3-(3-dimethylaminopropyl) carbodiimide (EDC), aniline, PEEK, sodium cyanoborohydride (NaBH_3_CN), boron trifluoride diethyl etherate (BF_3_·Et_2_O), trifluoroacetic acid (TFA), dimethylsulfoxide (DMSO), Tetrahydrofuran (THF), dichloromethane (DCM), and dimethylformamide (DMF) were purchased from Sigma Aldrich (Saint Louis, MO, USA). All the organic solvents were distilled under vacuum and stored with a Mark 4 Å molecular sieves. The American Type Culture Collection (ATCC, Manassas, VA, USA) provided osteoblast cells, whereas Gibco Invitrogen (Carlsbad, CA, USA) offered cell culture supplements and consumables. Promega (Madison, WI, USA) delivered CellTiter96^®^ AQueous One-Solution Cell Proliferation Assays.

### 2.2. Synthesis of Key Building Blocks

The pristine CNOs prepared by using a previously reported method as follows [33]. Carbon soot was deposited on a clean and dry glass slide over the flame of clarified butter (known as ghee in the Indian sub-continent region). The production yield of CNOs by this process depends on the amount of the clarified butter. A carboxylic acid functional group on CNOs was generated by a previously reported method [26].

(a) Synthesis of *N*-(4-aminophenyl)methacrylamide (APMA): *p*-Phenylenediamine (1.0 g, 9.25 mmol) was dissolved in anhydrous DCM, and triethylamine (1.3 mL, 9.25 mmol) was added. The reaction mixture was probe sonicated for 45 min. Next, methacryloyl chloride (903 μL, 9.25 mmol) was added dropwise into the above solution, and the probe sonication was continued for additional for 60 min. After completion of the reaction, the reaction mixture was washed with NaOH solution (two times) followed by water (two times) to reach a neutral pH. The organic phase containing the target compound (APMA) was collected and stored under MgSO_4_. Excess DCM was evaporated, and the crude product was purified by column chromatography using the mixture of ethyl acetate/hexane (2:1) with 2.0 vol% of trimethylamine as an eluent. Yield: 78%. ^1^H NMR (500 MHz, DMSO-*d*_6_) δ_ppm_ 9.3 (br, 1H), 7.31 (d, 2H), 6.6 (d, 2H), 5.8 (s, 1H), 5.4 (s, 1H), 4.9 (s, 2H), 2.0 (s, 3H); ^13^C NMR (125 MHz, DMSO-*d*_6_) δ_ppm_ 166.9, 144.6, 141.6, 128.2, 124.2, 119.9, 114.8, 18.1.

(b) Synthesis of poly (*N*-(4-aminophenyl)methacrylamide)) (PAPMA): A Schlenk tube (50 mL) was charged with 1.0 g of APMA (5.68 mmol), 1.0 wt% of, and 15 mL of anhydrous THF. Four freeze-pump-thaw cycles have performed to remove the oxygen. Then, the reaction mixture was stirred at 65 °C for 24 h under nitrogen atmosphere. Next, 100 mL of diethyl ether was added to the reaction mixture and the polymer was precipitated. The precipitated PAPMA was filtered and washed with an excess of DCM to discard the unreacted monomer and initiator. The obtained PAPMA was dissolved in methanol (~6 mL) followed by 50 mL of diethyl ether was added to re-precipitate. The re-precipitated PAPMA was purified and dried in the oven for 12 h at approximately 50 °C. The obtained PAPMA was characterized using gel permeation chromatography (GPC), FTIR, and NMR spectroscopy. ^1^H-NMR (500 MHz, DMSO-*d*_6_) δ_ppm_ 7.5 (br s, 2H), 6.4 (br s, 2H), 2.1 (br peak, 2H), 1.1–0.9 (br peak, 3H). ^13^C-NMR (125 MHz, DMSO-*d*_6_) δ_ppm_ 164.3, 153.4, 131.8, 124.5, 123.3, 121.9, 119.4, 116.8, 45.4, 18.7. GPC: Weight-average molecular weight (M_w_) = 34,679 and polydispersity index (PDI) = 1.32.

(c) Synthesis of PAPMA-CNOs (f-CNOs): 50 mg of COOH-CNOs, 285 mg of EDC, and 175 mg of NHS were dissolved in anhydrous DMF and probe sonicated for 60 min to activate the carboxyl groups of CNOs. 100 mg of PAPMA was added into the above mixture and probe sonicated for an additional 120 min. Then, the crude reaction mixture was centrifuged at 10,000 rpm to discard excess unreacted reagents and larger aggregates. The resulting solid was carefully washed with DMF/trimethylamine (9.9:0.1), and the black solid was dried under vacuum to obtain PAPMA-CNOs (f-CNOs). The obtained f-CNOs were characterized using NMR spectroscopy. ^1^H-NMR (500 MHz, DMSO-*d*_6_) δ_ppm_ 7.1 (br s, 2H), 6.5 (br s, 2H), 2.0–1.2 (br peak, 2H), 1.4–0.9 (br peak, 3H). ^13^C-NMR (125 MHz, DMSO-*d*_6_) δ_ppm_ 174.5, 167.5, 153.8, 131.8, 124.1, 123.2, 122.3, 119.1, 117.3, 44. 1, 18.9.

(d) Synthesis of Anilinated-PEEK (AN-PEEK): The synthesis was accomplished according to a previously reported protocol [34]. In a typical reaction, 1.0 g of PEEK was transferred into a mixture of anhydrous dichloromethane (40 mL) and trifluoroacetic acid (4 mL) and probe sonicated for 45 min to form a homogeneous solution. Then, 1.1 mL of aniline was added, followed by 0.5 mL of BF_3_·Et_2_O, which yielded an opaque solution. The reaction mixture was stirred for 48 h and then the mixture was poured into methanol. The resulting precipitate was filtered off under vacuum, and extracted with methanol. The resulting PEEK-imine (Schiff bases) was dried in a hot oven at 80 °C for overnight. Subsequently, 1.0 g of Schiff bases was dissolved in anhydrous DMSO and 1.1 g of NaBH_3_CN was added by portion-wise at 0 °C over 10 min. Then, the reaction mixture was stirred at 130 °C for 24 h. After completion of the reaction, the mixture was cooled to room temperature and filtered off. The solid product was dried in the oven at 80 °C after thoroughly rinsing with ethanol, and water. The synthesized polymer was denoted as “AN-PEEK”. ^1^H NMR (500 MHz, DMSO-*d*_6_) δ_ppm_ 7.7 (m, 2H), 7.5 (d, 4H), 7.0 (s, 4H), 6.9 (d, 4H), 6.8 (m, 1H), 6.5 (d, 2H), 4.9 (s, 1H). ^13^C-NMR (125 MHz, DMSO-*d*_6_) δ_ppm_ 159.6, 154.6, 136.9, 131.8, 128.8, 123.7, 118.0, 117.0, 114.1, 64.9.

### 2.3. Fabrication of AN-PEEK/f-CNOs Nanocomposite Thin Films

Nanofibers of AN-PEEK and f-CNOs were fabricated according to the previous protocols [28,30].

(a) AN-PEEK nanofibers: 2 wt% of AN-PEEK blending solution was prepared in DMF at 70 °C for 2 h. After obtaining a uniform solution, 2 mL of AN-PEEK blend solution was introduced in a dual orifice spinneret of Forcespinning^®^ (FS) and forcespun at 9000 rpm for 60 s. The obtained AN-PEEK nanofibers dried in a hot oven at 155 °C for 12 h. The temperature and relative humidity were optimized as 32 °C and 35–40%, respectively. The fibers prepared were denoted as AN-PEEK fibers. Similarly, 5 mg of DOX was loaded into the above AN-PEEK blending solution to fabricate DOX loaded AN-PEEK fibers, which labelled as DOX/AN-PEEK fibers.

(b) f-CNOs nanofibers: 1 wt% of f-CNOs was prepared in DMF at room temperature for 2 h. After attaining a uniform solution, 2 mL of f-CNOs blend solution was fed in a dual orifice spinneret of FS and forcespun at 9000 rpm for 60 s. The obtained AN-PEEK nanofibers dried in a hot oven at 155 °C for 12 h. The optimum temperature and relative humidity were found to be 32 °C and 35–40%, respectively. The fibers prepared were denoted as f-CNOs fibers. Similarly, 5 mg of DOX was loaded into the above f-CNOs blending solution to fabricate DOX loaded f-CNOs fibers, which represented as DOX/f-CNOs fibers.

(c) Preparation of AN-PEEK/f-CNO thin films: AN-PEEK/f-CNOs thin films were fabricated via vacuum-assisted LbL assembly followed by a hot pressing approach using a modified protocol of previous report [35]. For this, AN-PEEK fibers were cut into small pieces and AN-PEEK fiber dispersion (pH 1.5) ultra-sonicated for 60 min was vacuum filtered onto a porous nylon membrane (0.1 µm pore size) for 5 min to obtain a wet viscoelastic AN-PEEK layer. Then, the DOX/f-CNOs fiber dispersion (pH 12) was added into the filter and filtrated for 30 min. Likewise, AN-PEEK and DOX/f-CNOs fiber dispersions have alternatively filtered to generate LbL architecture. This cycle was repeated *n* times to produce vacuum-assisted LbL assembled [AN-PEEK/f-CNOs]*_n_* thin films, which were rinsed with DI water to discard impurities. Then, the filter membranes were hot-pressed at 1 MPa and 120 °C for 2 h. Finally, the thin films were peeled off from the membrane. A series of ultra-flexible AN-PEEK/f-CNOs thin films were fabricated with 1.5, 2.5, 5, 7.5, and 10 wt% of f-CNOs. Hereafter, AN-PEEK/f-CNOs (1.5 wt%), AN-PEEK/f-CNOs (2.5 wt%), AN-PEEK/f-CNOs (5 wt%), AN-PEEK/f-CNOs (7.5 wt%), and AN-PEEK/f-CNOs (10 wt%) composites denoted as 1.5, 2.5, 5, 7.5, and 10% composite thin films.

### 2.4. In Vitro Drug Release Measurements

A pH-responsive drug (DOX) release from AN-PEEK and AN-PEEK/f-CNOs thin films was achieved by using UV-spectrophotometer (Agilent Technologies, 89090A, Santa Clara, CA, USA). Around 50 mg of thin films were submersed in DMEM (pH 4.5, 6.5, and 7.4) followed by incubating at 37 °C. After predetermined time intervals, 2 mL of DOX-released solution was withdrawn and replenished with an equal amount of fresh medium to record DOX absorption intensity. The release and concentration of DOX were recorded on a UV spectrophotometer at 480 nm. A drug release study was performed in triplicates. The drug release (%) was calculated from the following formula:

(1) $$\mathrm{Drug}\;\mathrm{release}\;(\%)\;=\;\frac{Mass\;of\;drug\;loaded\;films-Mass\;of\;drug\;released\;}{Mass\;of\;drug\;released\;}\times 100$$

### 2.5. Characterizations of AN-PEEK/f-CNOs Nanocomposite Thin Films

UV-vis measurements have taken by using UV-spectrophotometer (Agilent Technologies, 89090A). Scanning Electron Microscopy (SEM, ZEISS EVO^®^ MA 25, Ostalbkreis, Baden-Württemberg, Germany) was used to measure the morphological properties of composite films. Fourier transform infrared (FTIR) spectroscopy (Perkin Elmer Universal ATR Sampling Accessory Frontier, Waltham, MA, USA) was used in the wavenumber range between 4000 to 500 cm^−1^. Raman spectroscopy (Renishaw inVia Raman microscope with a green laser of 514 nm) was used to measure the vibrational and rotational modes of the samples. Thermal gravimetric analysis (TGA, SDT Q600, TA Instruments) was used to study the mass loss of the samples. The heating rate was 10 °C/min under a nitrogen atmosphere at a temperature range of 20 °C to 800 °C. A tensile-testing machine (Instron 3365, Instron, Norwood, MA, USA) was used to measure the mechanical properties of composites films. The freestanding composite strips were cut to 1 ± 0.3 mm of width. The rate of stretching was 0.01 mm/s, and the initial gap between grips was 4 ± 1 mm. All the testing dimensions were directly measured by precision calipers for accuracy, and the tensile readings were reproduced and averaged 5 times. A profilometer (P-6, KLA-Tencor, Milpitas, CA, USA) was used to record the thickness and toughness of the composite films. Malvern Nano-ZS instrument was utilized to measure hydrodynamic size, polydispersity indexes (PDIs), and ζ-potentials of specimens. Phosphate Buffered Saline (pH 7.4) was used to disperse the specimens. The size, as well as ζ-potential of the specimens were measured and dynamic light scattering (DLS) tests were run in triplicate.

### 2.6. Cytotoxicity Evaluation of AN-PEEK/f-CNOs Nanocomposite Thin Films

In vitro biocompatibility of the nanocomposite films was evaluated with human osteoblast cells. The cytotoxicity of f-CNOs was measured by culturing bone-forming cells in a medium with different concentrations of f-CNOs and measuring cell viability. Initially, composite specimens were sterilized, and osteoblast cells (4.8 × 10^5^ cells/cm^2^) were planted on the surface of sterilized specimens, which was nurtured in DMEM with 5% CO_2_ atmosphere at 37 °C. For this, 100 μg/mL penicillin, 5% (*w/v*) fetal bovine serum, and 100 μg/mL streptomycin were used to supplement the DMEM. Non-adherent cells were discarded on the next day and osteoblasts planted specimens were shifted into new wells. Then, 20 µL of CellTiter was added over the specimens on day 1 to day 3 and nurtured for 1 h under dark conditions. The absorbance of each well was measured using a microplate reader (Synergy HT, BioTek, Winooski, VT, USA). The cell viability of the specimens was calculated from the absorbance of each well. A blank tissue culture plate was a controller and viability measurements were run in triplicate.

### 2.7. Statistical Analysis

All the quantitative data were expressed as mean ± standard deviations with *n* = 3. Statistical analysis was performed by one-way analysis of variance (ANOVA) followed by Tukey’s post hoc tests using Minitab17 (Minitab, State College, PA, USA) and statistical significance was considered at *p* ≤ 0.05.

## 3. Results and Discussion

**Scheme 1:**
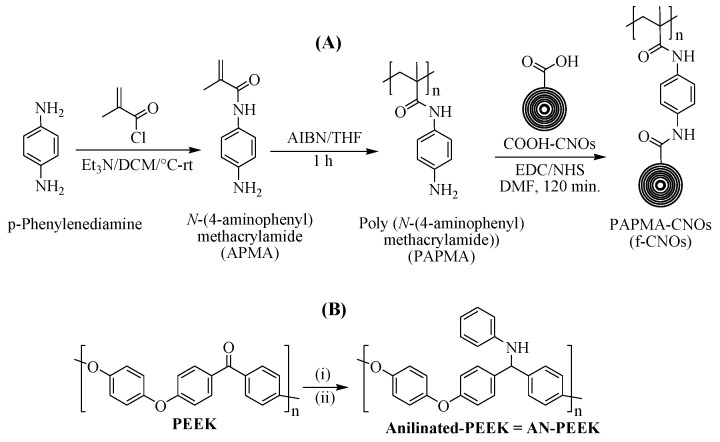
(**A**) Synthetic route of PAPMA-CNOs (f-CNOs) from p-phenylenediamine. (**B**) Synthetic route of anilinated-poly (ether ether ketone) (AN-PEEK) from PEEK and aniline (AN). (i) AN, CH_2_Cl_2_/CF_3_COOH, BF_3_.Et_2_O, 48 h, (ii) NaBH_3_CN, DMSO, 130 °C, 24 h.

The APMA monomer is a crucial part of designing an amide polymer containing amine functionality at the para position of phenyl ring for the coupling of COOH-CNOs. APMA monomer was synthesized by reacting *p*-phenylenediamine with methacryloyl chloride and TEA using DCM solvent under the N_2_ atmosphere at 0 °C to room temperature (Scheme 1A). The chemical structure of APMA was confirmed by NMR spectra (Supplementary Materials Figure S1). The resonance of olefinic protons of methacryloyl moiety was observed at δ 5.8 (singlet) and δ 5.4 (singlet), whereas the amide proton (CONH) was detected at δ 4.9 as a singlet (Supplementary Materials Figure S1a). Besides, two doublets of phenyl ring were observed at 7.3 and 6.6 ppm, respectively. Whereas the free amine group at the para position of the phenyl ring was located as a broad peak at δ 9.3 (Figure S1a). The homo-polymerization of APMA was initiated by using 1 wt% of AIBN in THF at room temperature under an N_2_ atmosphere for 60 min. NMR spectra described the molecular structure of PAPMA, and the resonance peaks of all the protons of PAPMA were readily assignable. Two singlets of methacryloyl moiety at δ 5.8 and δ 5.4 (Supplementary Materials Figure S1a) were completely disappeared, and the broad peaks of PAPMA were detected in the range of 2.5 to 1.2 ppm (Supplementary Materials Figure S2a), which confirmed the complete transformation of monomer into homopolymer (PAPMA, Supplementary Materials Figure S2). Next, COOH-CNOs were reacted with PAPMA in the presence of EDC/NHS to obtain covalently attached PAPMA-CNOs (Scheme 1A); hereafter PAPMA-CNOs are denoted as f-CNOs. NMR analysis was used to characterize the molecular structure of f-CNOs (Supplementary Materials Figure S3). Notably, the ^13^C NMR spectrum of f-CNOs showed a new amide peak at δ 174.5, which confirms the covalent attachment of COOH-CNOs with PAPMA via amidation reaction (Supplementary Materials Figure S3b). Further, molecular weight (M_w_) of PAPMA was measured by GPC analysis, which exhibited 34,679 of M_w_ and 1.32 of polydispersity index (Supplementary Materials Figure S4). The f-CNOs dispersions were prepared in DMF, DMSO, and Dulbecco’s Modified Eagle’s Medium (DMEM); the f-CNOs dispersions were highly stable for more than 12 months.

Aniline moiety was introduced on PEEK molecular surface via a two-step reaction to improve the solubility and adhesion properties of PEEK (Scheme 1B). In the first step of the PEEK modification, polymer stabilization was the Schiff base reaction of pristine PEEK with aniline, promoted by the mild Lewis acid BF_3_·Et_2_O in DCM/TFA at room temperature. The Schiff base product was obtained within 48 h, and subsequently, PEEK-imine was reduced by using NaBH_3_CN in anhydrous DMSO at 130 °C to provide anilinated PEEK (AN-PEEK). The expected final product of this reaction (AN-PEEK) was confirmed by spectroscopic characterizations (FTIR, ^1^H NMR, and ^13^C NMR). As shown in Supplementary Materials Figure S5a, a clear singlet signal of benzhydryl proton has appeared at δ 4.9. Besides, a ketone characteristic peak corresponding to δ 200 ppm of pristine PEEK was disappeared, and a new peak of benzhydryl carbon was observed at δ 64.9 in ^13^C NMR of AN-PEEK (Supplementary Materials Figure S5b). This confirms the conversion of carbonyl functionality of pristine PEEK into AN-PEEK, which was further confirmed by FTIR.

After the synthesis of key building blocks, AN-PEEK, and f-CNOs nanofibers were fabricated using FS, and continuous and homogeneous nanofibers were obtained (Supplementary Materials Figure S6). FS is a versatile technique, which produces high throughput nanofibers from a wide range of materials including non-conductive and conductive polymers [36]. Several forcespun nanofibers including PCL, BSA, gelatin/poly (epichlorohydrin-co-ethylene oxide), gelatin/zein, and gelatin/polylactic acid) have manufactured by FS and used for tissue engineering and drug release applications [28,30,37,38,39]. Then, an LbL technology was employed to prepare AN-PEEK/f-CNOs nanocomposite thin films. AN-PEEK fibers dispersed in pH 1.5 solution was LbL assembled with f-CNOs fibers following procedure demonstrated in the experimental section. The f-CNOs colloids were negatively charged due to the pH 12, whereas the AN-PEEK was positively charged under the employed conditions of LbL assembly. The driving force of f-CNOs adsorption on AN-PEEK was a manifold of impartially weak interactions, including van der Waals attraction, hydrophobic interaction, dipolar electrostatic forces, π–π-stacking, and hydrogen bonding. The recurrence of LbL cycles ensued in linear film growth, and the growth rate was dependent on the pH of dispersion solutions. It is well known that homogeneous dispersion of nanofillers can improve the mechanical properties of nanocomposites when they are incorporated into composites due to the sufficient stress transfer with a polymer matrix [40].

To investigate this perception, the colloidal dispersion of f-CNOs and AN-PEEK/f-CNOs with different wt% of f-CNOs (1.5, 2.5, 5, 7.5, and 10%) in water or buffer was realized by the DLS measurements. The hydrodynamic size, PDI, and ζ-potential values of samples were investigated (Table 1). Pristine f-CNOs presented 113 ± 1.14 nm of size and −37.3 ± 0.45 of ζ-potential values, respectively, which impedes agglomeration and signifies its colloidal stability. The smallest PDI of 0.15 ± 0.03 confirms the homogeneous colloidal stability f-CNOs, whereas 1.5% of nanocomposite exhibited slightly increased diameter (123 ± 7.14 nm) with reduced ζ-potential values (−5.1 ± 1.05). The smallest PDI of 0.19 ± 0.03 confirmed the uniform distribution of f-CNOs within the polymer matrix.

Thus, by increasing wt% of f-CNOs, the size of nanocomposites was improved and the ζ-potential of nanocomposites was significantly enriched, which confirmed that f-CNOs existed in the negatively charged form under the employed conditions. For instance, 5% of the nanocomposite displayed slightly enhanced hydrodynamic size (151 ± 3.81 nm), and ζ-potential value (−13.1 ± 1.03). However, 0.23 ± 0.04 of the PDI of f-CNOs signified its colloidal stability and homogeneous distribution, which confirms the electrostatic interactions including π–π-stacking, and hydrogen bonding between f-CNOs and AN-PEEK. On the other hand, 10% of the nanocomposite showed around 193 ± 3.31 nm of size and −23.4 ± 1.17 of ζ-potential with 0.39 ± 0.09 of PDI. Overall, the DLS experiments confirmed the uniform distribution of f-CNOs (<5%) within AN-PEEK matrixes.

**Figure 1:**
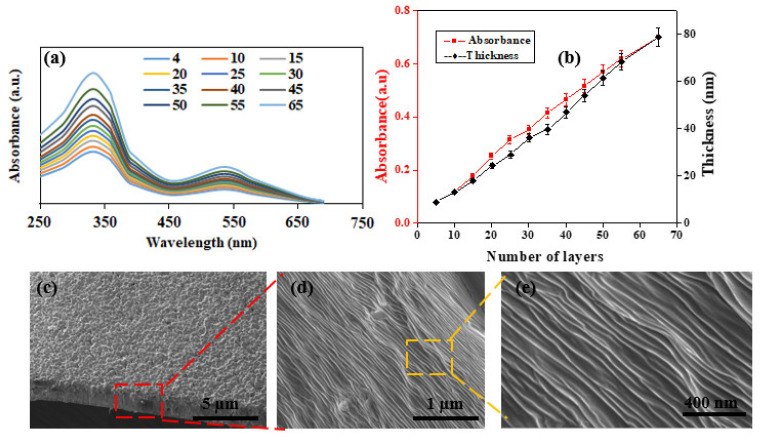
(**a**) UV-vis spectra of AN-PEEK/f-CNOs LbL films as a function of the number of layers. (**b**) Absorbance at 340 nm as a function of the number of layers and thickness of AN-PEEK/f-CNOs thin films measured by profilometry. (**c**) Cross-sectional, and (**d**,**e**) side-view of the SEM images of AN-PEEK/f-CNOs nanocomposite thin film with 5 wt% of f-CNOs.

**Figure 2:**
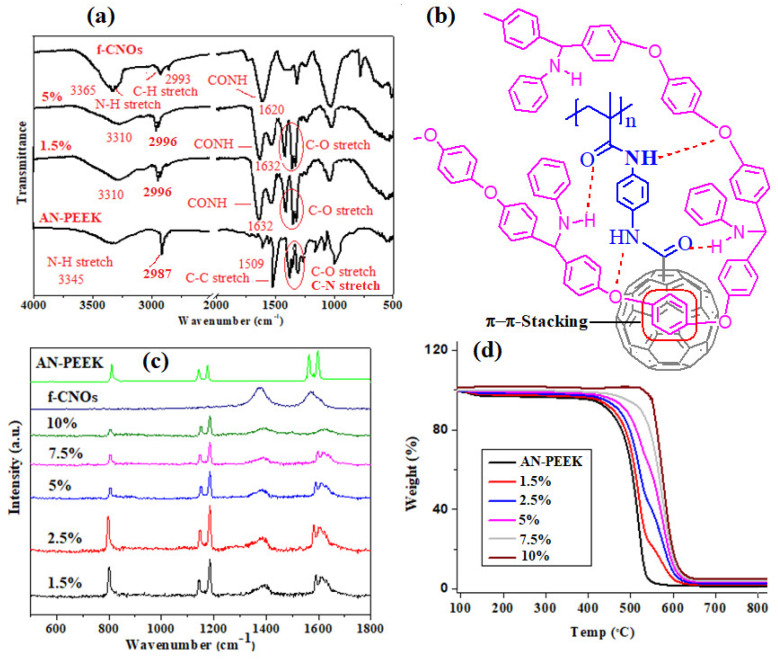
(**a**) FTIR of AN-PEEK and AN-PEEK/f-CNOs composite films. (**b**) Molecular structure of AN-PEEK/f-CNOs complex presenting the possible π–π-stacking between AN-PEEK, and f-CNOs and hydrogen bonding interactions between AN-PEEK, and PAPMA moiety. (**c**) Raman, and (**d**) TGA curves of AN-PEEK and AN-PEEK/f-CNOs nanocomposite films. The percentage fractions in the plots denotes wt% of the f-CNOs content in the AN-PEEK.

Next, UV-visible spectroscopy and profilometry were utilized to scrutinize the growth behavior of the AN-PEEK/f-CNOs LbL films. The gradual increase in the UV-visible absorbance at around 340 nm, demonstrated linear growth of the AN-PEEK/f-CNOs LbL films with an increasing number of layers (Figure 1a,b (red line)). The linear growth was also established by profilometry (Figure 1b). The growth factor of (AN-PEEK/f-CNOs)*_n_* multilayers is almost linear with the first 65 layers, reaching a film thickness of about 76 nm (Figure 1b (black line)), which provides a thickness increment for each layer of 1.2 nm. The composite films attributed to form primarily hydrogen bonding and aromatic (π–π) stacking between multilayer components (Figure 2b). Furthermore, van der Waals interfaces are also anticipated to contribute to the mechanical integrity of the composite thin film.

The AN-PEEK/f-CNOs are uniformly packed on the surface of the substrate during the LbL assembly (Figure 1c). SEM analysis exhibits that the surface of thin films is fairly flat (Figure 1c), and the fracture edges of the films show a well-stacked layer structure through the entire cross-section (Figure 1d,e). These results reveal that f-CNOs can be assembled to form highly ordered macroscopic structures under LbL-induced assembly. By adjusting the volume of the colloidal dispersion, the thickness of the film samples can be varied from the tens of nanometers to 5 µm. Nonetheless, only stable and agglomeration-free f-CNOs colloids can produce uniform and smooth thin films. As can be seen, the AN-PEEK/f-CNOs nanocomposite films display a multilayered structure composed of the partially embedded f-CNOs layer and AN-PEEK substrate layer, and the surface of f-CNOs becomes considerably coarser. During the LbL assembly, the f-CNOs perhaps wrapped by the AN-PEEK owing to the extensive hydrogen-bonding interactions between the carbonyl group of PAPMA on CNOs surface and the amino group of AN-PEEK as well as aromatic stacking between CNOs and AN-PEEK. Subsequently, the hot-pressing process was promoted further embedding of f-CNOs into the AN-PEEK substrate, which causes the densification and desiccation of the AN-PEEK/f-CNOs films, attaining the ultrathin films with a multilayered structure. Importantly, the densely wrapped f-CNOs can assent mechanical stress from the polymer matrix, which can improve the mechanical properties of nanocomposites.

To determine the physicochemical interactions of thin films, FTIR analysis was performed for AN-PEEK and AN-PEEK/f-CNOs films with 1.5 wt%, and 5 wt% of f-CNOs. The remaining composite films resembled the aforementioned composites, thus we did not include in Figure 2a. An FTIR characteristic peak at 1651 cm^−1^ assigned to C=O of pristine PEEK was disappeared, and a characteristic peak for the C-N aliphatic vibrational band was detected in the range of 1250–1300 cm^−1^ from the aliphatic proton of benzhydryl moiety (Figure 2a). A characteristic peak for the C−H aliphatic vibration appeared around 2987 cm^−1^ from the aliphatic proton of the benzhydryl carbon, which was further confirmed by NMR (Supplementary Materials Figure S5).

On the other hand, f-CNOs shown the main characteristic peaks at 3365, 2993, and 1620 cm^−1^ for N-H, C-H stretching, and the carbonyl group of the amide (CONH), respectively (Figure 2a). After the fabrication of composite films, the characteristic peaks of AN-PEEK/f-CNOs were shifted to different frequencies. For instance, a characteristic shoulder band was noticed at 1610 cm^−1^ close to the peak at 1632 cm^−1^ (C=O), and a characteristic peak of N−H was detected at 3310 cm^−1^, which is lower than that in the pristine AN-PEEK, and f-CNOs. The presence of the shoulder peak and shift of the N−H peak to the lower frequency reveal the formation of hydrogen bonding interactions between AN-PEEK, and PAPMA on CNOs (Figure 2b).

In addition, the aromatic molecular structures within AN-PEEK may also provide π–π interactions with CNOs (Figure 2b). The fabrication of interfacial regions with strong interactions is a key factor for the achievement of enhanced strength and stiffness within nanocomposites [40]. It is hypothesized that the multiple hydrogen bonds and π–π interactions conceivably tolerate the strengthening and stress transfer from AN-PEEK to f-CNOs, which may result in high strength and Young’s modulus. Furthermore, Raman spectroscopy was utilized to appraise other possible interactions. Two main characteristic graphite peaks of f-CNOs were observed at 1357 cm^−1^ (D band) and 1581 cm^−1^ (G band) assigned to the disorder in carbon systems, and in-plane vibration of the C-C band, respectively (Figure 2c). However, the characteristic bands at 1590, and 1604 cm^−1^ (C-C stretching of AN-PEEK) were gradually shifted to a higher frequency (1613 cm^−1^) upon the addition of more f-CNOs. At high content of f-CNOs (10%), two bands (1590, and 1604 cm^−1^ of AN-PEEK) were completely disappeared or broadened (Figure 2c), which indicates the π–π stacking interactions between aromatic rings of AN-PEEK, and CNOs.

As shown in Figure 2d, the thermal decomposition and stability of thin films were scrutinized by using a TGA analysis. Specifically, pristine AN-PEEK exhibited initial (T_0.1_) decomposition temperature at approximately 425 °C, whereas midpoint (T_0.5_) degradation temperature at around 548 °C. On the other hand, AN-PEEK/f-CNOs thin films demonstrated augmented thermal stability and significant weight losses observed in a range of 517–621 °C (Figure 2d). These results validated that both thermal degradation, as well as onset temperature of AN-PEEK/f-CNOs thin films, have amended. The thermal stability of thin films was f-CNOs dose-dependent. The high thermal stability of composite films would enhance the mechanical properties [35].

**Figure 3:**
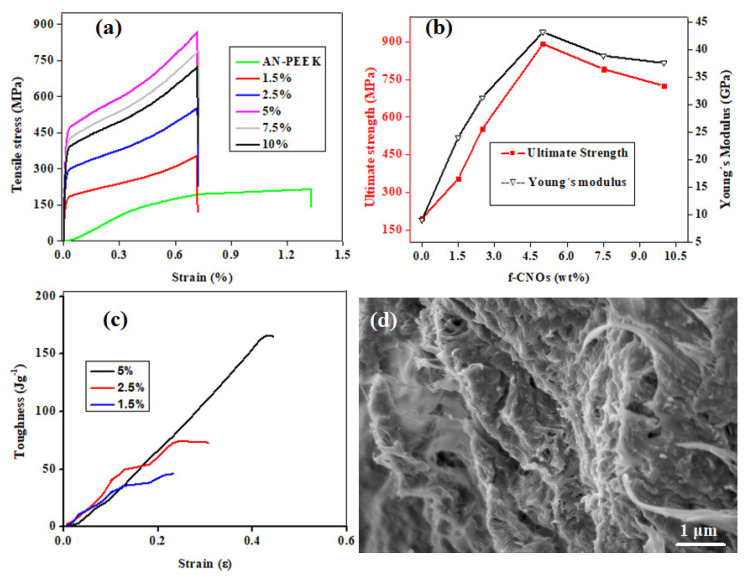
Quantitative mechanical characterization of AN-PEEK/f-CNOs composite films. (**a**) Stress–strain, (**b**) ultimate strength-Young’s modulus, (**c**) toughness-strain curves for nanocomposite thin films obtained from uniaxial tensile. (**d**) SEM images of fracture surfaces of AN-PEEK/f-CNOs with 5 wt% of f-CNOs, prepared from tearing tests on notched samples. The weight fraction in the *x*-axis (**b**) and the percentage in plots (**a**,**c**) represent the wt% of f-CNOs content in the AN-PEEK.

The mechanical properties of AN-PEEK and nanocomposite films with various wt% of f-CNOs were measured and presented in Figure 3. AN-PEEK thin film exhibited a tensile strength (σ) of 195.4 ± 1.1 MPa and Young’s modulus (E) of 9.9 ± 0.3 GPa (Figure 3a,b). The inclusion of f-CNOs has obviously reduced the ultimate strain of AN-PEEK film (Figure 3a). The 1.5 wt% addition of f-CNOs amended σ to 354.1 ± 3.5 MPa and E to 24.1 ± 0.8 GPa (Figure 3a,b). The 2.5 wt% composite films exhibited in further enhancement of σ to 552.9 ± 6.1 MPa and E to 31.3 ± 0.4 GPa (Figure 3a,b), whereas AN-PEEK/f-CNOs with 5 wt% of f-CNOs significantly improved σ to 891.4 ± 8.2 MPa and E to 43.2 ± 1.1 GPa (Figure 3a,b). This improvement corresponded to 356.2 % and 336.4 % for σ and E compared with AN-PEEK thin film, respectively. Further increment of f-CNOs content resulted in the decrease of both σ and E (Figure 3a,b), this perhaps being due to the agglomeration of f-CNOs at the higher content (i.e., 7.5 and 10 wt%). Note that the functionalization of the CNOs surface affected not only the stress transfer function but also the overall contents of f-CNOs in the composite films.

Additionally, the toughness of 1.5, 2.5, and 5 wt% of LbL nanocomposite films was measured (Figure 3c). The toughness of LbL films was measured to be 43.5, 74.0, and 164.5 Jg^−1^ for 1.5, 2.5, and 5 wt%, respectively. These toughness values reflected the significant effects of simultaneous changes in f-CNOs content and stress transfer circumstances at the interface between f-CNOs and AN-PEEK. Thus, the variances in ultimate tensile strain and toughness among nanocomposite films can be indorsed to their diverse capabilities to reorganize under tensile stress. It is worth mention that widespread interfacial interactions between nanoscale materials are essential in biological tissues and are responsible for many of their functionalities [41]. The method of nanocomposite fabrication described here exploits nanomaterials interaction that leads to the diverse behaviors of AN-PEEK/f-CNOs thin films.

We examined the fracture area under the tensile load to investigate the crack/fracture process under the tensile test. As shown in Figure 3d, the films exhibited a uniform and dense microstructure. AN-PEEK/f-CNOs showed a layered structure composed of reinforced f-CNOs and AN-PEEK layers. A fracture area with some broken microstructures revealed not only AN-PEEK but also f-CNOs undergo elongation, pullout, and breakage. The superior mechanical properties of the nanocomposite structural assemblies processed by AN-PEEK could be advantageous over traditional polymers in single molecular conditions. The noncovalent interactions between AN-PEEK and f-CNOs led to symbiotic interchange and self-organization performance under tensile stress. The hydrogen-bonded PAPMA chains bridged the AN-PEEK network and facilitated load transfer through the AN-PEEK. Characteristic orientation could be observed at the tear surface area of nanocomposite film (Figure 3d). Overall, these enhanced mechanical properties of thin films were achieved by the multiple electrostatic interactions between AN-PEEK and f-CNOs, which delivered a strong bonding interface.

**Figure 4:**
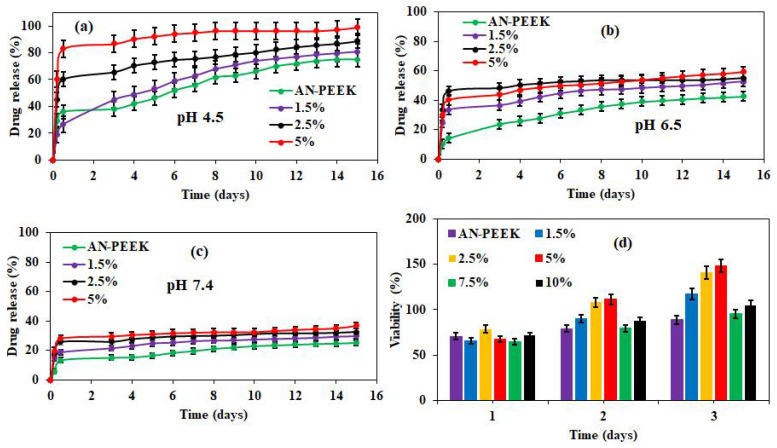
Drug release results of DOX-loaded AN-PEEK and AN-PEEK/f-CNOs thin films: (**a**) pH 4.5, (**b**) pH 6.5, and (**c**) pH 7.4 at 37 °C in DMEM solution. (**d**) Cell viability of thin films.

The mechanical and structural properties are important factors for scaffolds to mimic the structure of the native extracellular matrix (ECM) of cells [42]. It is known that the pH values of cancer cells (pH 4.5–6.5) differ from normal cells (pH 7.4) [43,44]. Therefore, in vitro drug release from DOX loaded AN-PEEK and AN-PEEK/f-CNOs composite films was measured at pH 4.5, 6.5, and 7.4 (Figure 4). DOX loaded AN-PEEK films presented a slight burst release followed by sustained release (75.1%) at pH 4.5 in 15 days (Figure 4a). AN-PEEK/f-CNOs composite film with 1.5 % f-CNO showed around 81.2% of drug release at pH 4.5.

In contrast, the amount of DOX release from AN-PEEK/f-CNOs composite film with 2.5% f-CNO was 88.1% at pH 4.5 (Figure 4a). Further, AN-PEEK/f-CNOs composite film with 5% f-CNO displayed around 99.2% at pH 4.5. The results indicated that the drug release from composite films has significantly affected by environmental pH. We further explore whether the variation of pH value can influence the drug release profile of composite thin films. Under pH 6.5, AN-PEEK, 1.5%, 2.5%, and 5% composite thin films exhibited 42.6, 52.6, 55.1, and 59.3% of release, respectively (Figure 4b). Besides, we employed the drug release profile under physiological pH 7.4 (Figure 4c). AN-PEEK film showed around 25.5% of drug release over 15 days, whereas, 1.5%, 2.5%, and 5% composite thin films presented 29.7, 32.4, and 36.8% of release, respectively (Figure 4c). The results reveal that under physiological environment (pH 7.4); DOX molecules strongly bound on the surface of f-CNTs due to the π–π stacking between f-CNOs and DOX molecules. Overall, the drug release rate of composite films at pH 4.5 was higher than that at pH 6.5 and 7.4. This could be due to the protonation of the–NH_2_ group on the surface of DOX, which weakens the electrostatic interactions between f-CNOs and DOX molecules. Therefore, maximum drug release (99.2%) was observed under an acidic environment (pH 4.5) from the thin films (Figure 4a). Thus, AN-PEEK/f-CNOs nanocomposite film with the highest content of f-CNOs (5%) remained extremely sensitive at pH 4.5.

Next, we investigated the cytotoxicity of nanocomposite thin films. For this, human osteoblasts have nurtured on the surface of AN-PEEK and AN-PEEK/f-CNOs composite films (Figure 4d). On day one, AN-PEEK and AN-PEEK/f-CNOs nanocomposite films exhibited comparable cell viability. On the second day, 2.5% and 5% nanocomposite films showed slightly upgraded cell viability. While AN-PEEK and control (culture plate) showed comparable cell viability. Interestingly, on the third day, 2.5% and 5% nanocomposite films exhibited a substantial enhancement in the viability (Figure 4d). This could be due to the efficient roughness, homogeneous colloidal dispersion, or uniform distribution of f-CNOs within the polymer matrix of 2.5% and 5% nanocomposite films. Besides, analogous cell viability has observed in AN-PEEK and remaining composite films (Figure 4d). These results revealed that the AN-PEEK/f-CNOs composite films with unique cytocompatibility would be potential drug vehicles. It was theorized that good cytocompatibility and superior mechanical properties of composite films could be the most compelling factors for nanocomposites in materials science.

## 4. Conclusions

Functionalized AN-PEEK and f-CNOs synthesized and nanofibers have primed using Forcespinning for the first time ever. AN-PEEK/f-CNOs composite thin films fabricated by the LbL strategy showed enhanced mechanical properties. We demonstrated that the multiple interfacial interactions, high content of f-CNOs, and stress transfer between AN-PEEK and f-CNOs are the key factors that impelling the mechanical properties of layered composite films. The described LbL method eases the structural defects ascending from phase segregation. The composite films were successfully extended for the pH-responsive sustained drug release and around 99% of drug release was measured over 15 days at pH 4.5. The cytotoxicity studies showed good cell viability on the surface of LbL composite thin films. Nevertheless, the current thin films would be potential nanocomposites for biomedical applications.

## Figures and Tables

**Table 1:** The size, PDI, and zeta potential of f-CNOs ^a^.

Si. No.	Sample Name	Size (nm)	PDI	ζ-Potential
1.	f-CNOs	113 ± 1.14	0.15 ± 0.03	−37.3 ± 0.45
2.	1.5%	123 ± 7.14	0.19 ± 0.03	−5.1 ± 1.05
3.	2.5%	138 ± 4.14	0.21 ± 0.01	−9.3 ± 1.47
4.	5%	151 ± 3.81	0.23 ± 0.04	−13.1 ± 1.03
5.	7.5%	167 ± 5.19	0.29 ± 0.12	−20.6 ± 0.31
6.	10%	193 ± 3.31	0.39 ± 0.09	−23.4 ± 1.17

^a^ The percentage fraction in the table denotes the wt% of f-CNOs content in the AN-PEEK composites.

## Supplementary Materials

The following are available online at https://www.mdpi.com/1999-4923/12/12/1208/s1, Figure S1. (**a**) ^1^H-NMR, (**b**) ^13^C-NMR spectra of APMA, Figure S2. (**a**) ^1^H-NMR, (**b**) ^13^C-NMR spectra of PAPMA, Figure S3. (**a**) ^1^H-NMR, (**b**) ^13^C-NMR spectra of PAPMA-CNOs, Figure S4. Gel permeation chromatography (GPC) curve of PAPMA, Figure S5. (**a**) ^1^H-NMR, (**b**) ^13^C-NMR spectra of AN-PEEK, Figure S6. SEM micrographs of (**a**) AN-PEEK and (**b**) f-CNOs nanofibers.
