# Supplementary material for: Carbon Nano-Onions Reinforced Multilayered Thin Film System for Stimuli-Responsive Drug Release
Source: Pharmaceutics. 2020 Dec 13;12(12):1208. doi: 10.3390/pharmaceutics12121208 (PMC7764530; doi:10.3390/pharmaceutics12121208)
Supplement: Supplementary file 1 [file pharmaceutics-12-01208-s001.pdf]

pharmaceutics

MDPI

Supplementary Material: Carbon Nano-Onions Reinforced Multilayered Thin Film System for Stimuli-Responsive Drug Release

Narsimha Mamidi, Ramiro Manuel Velasco Delgadillo, Aldo Gonzáles Ortiz and Enrique V. Barrera

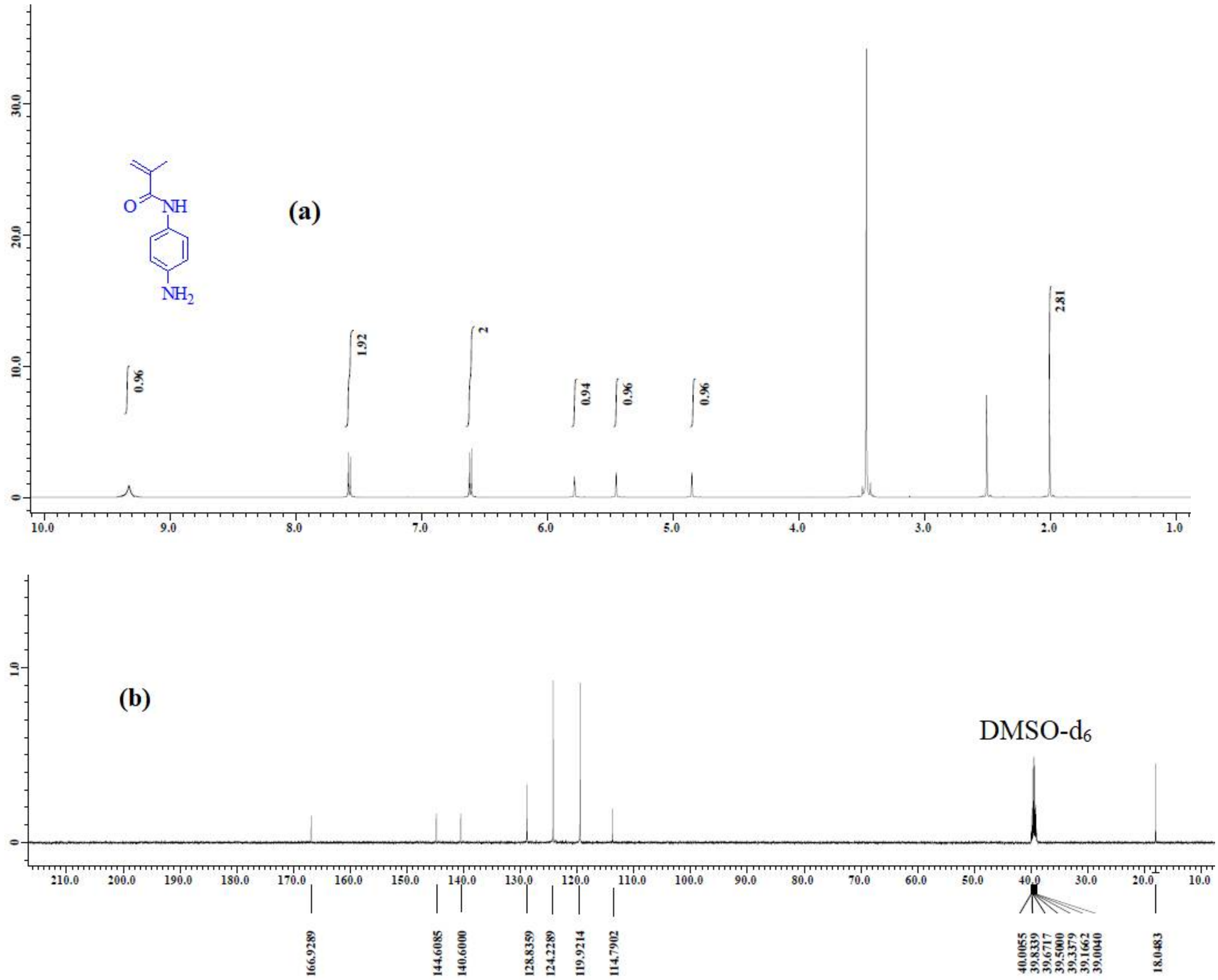

Figure S1. (a) ^{1}H-NMR, (b) ^{13}C-NMR spectra of APMA.

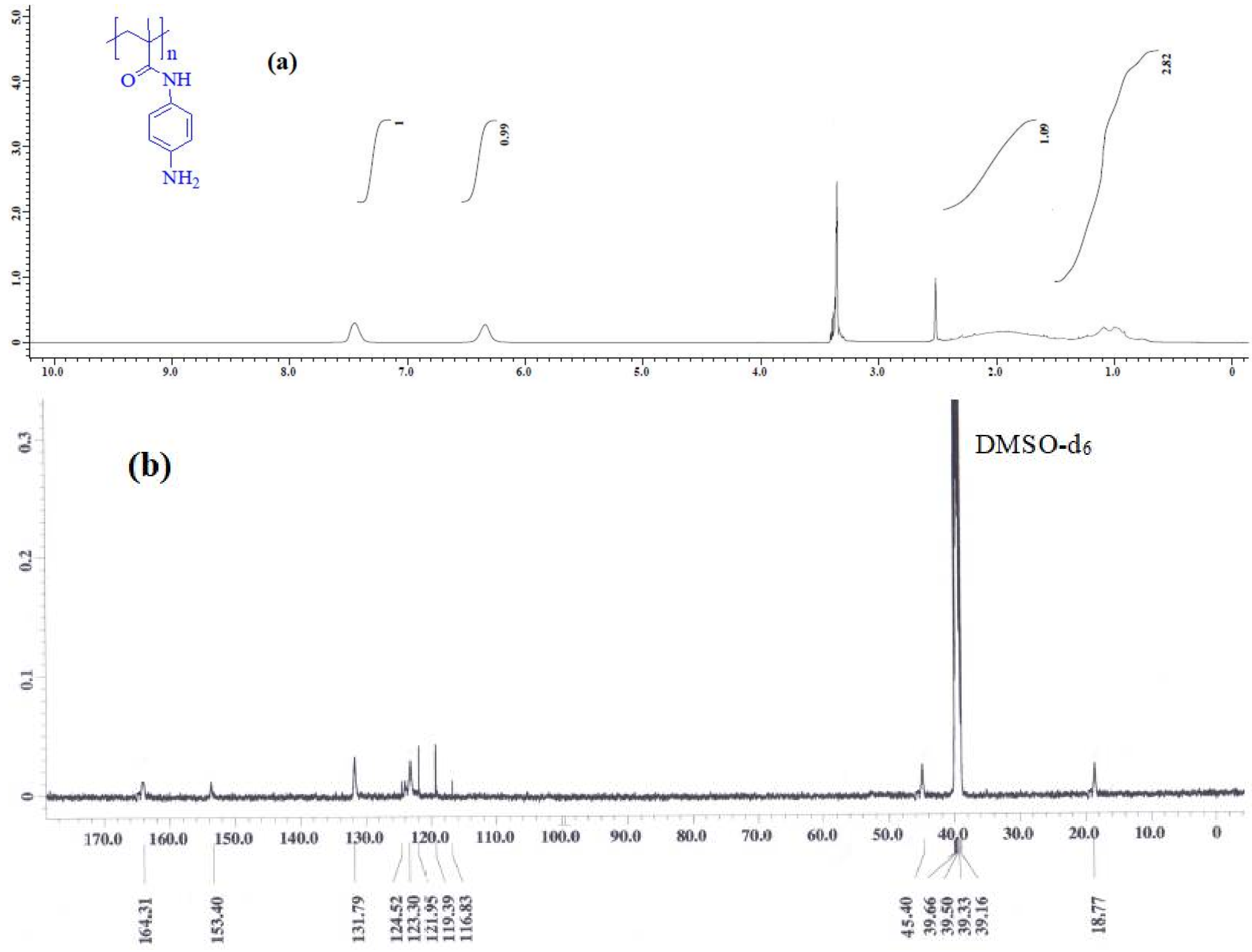

Figure S2. (a) ^{1}H-NMR, (b) ^{13}C-NMR spectra of PAPMA.

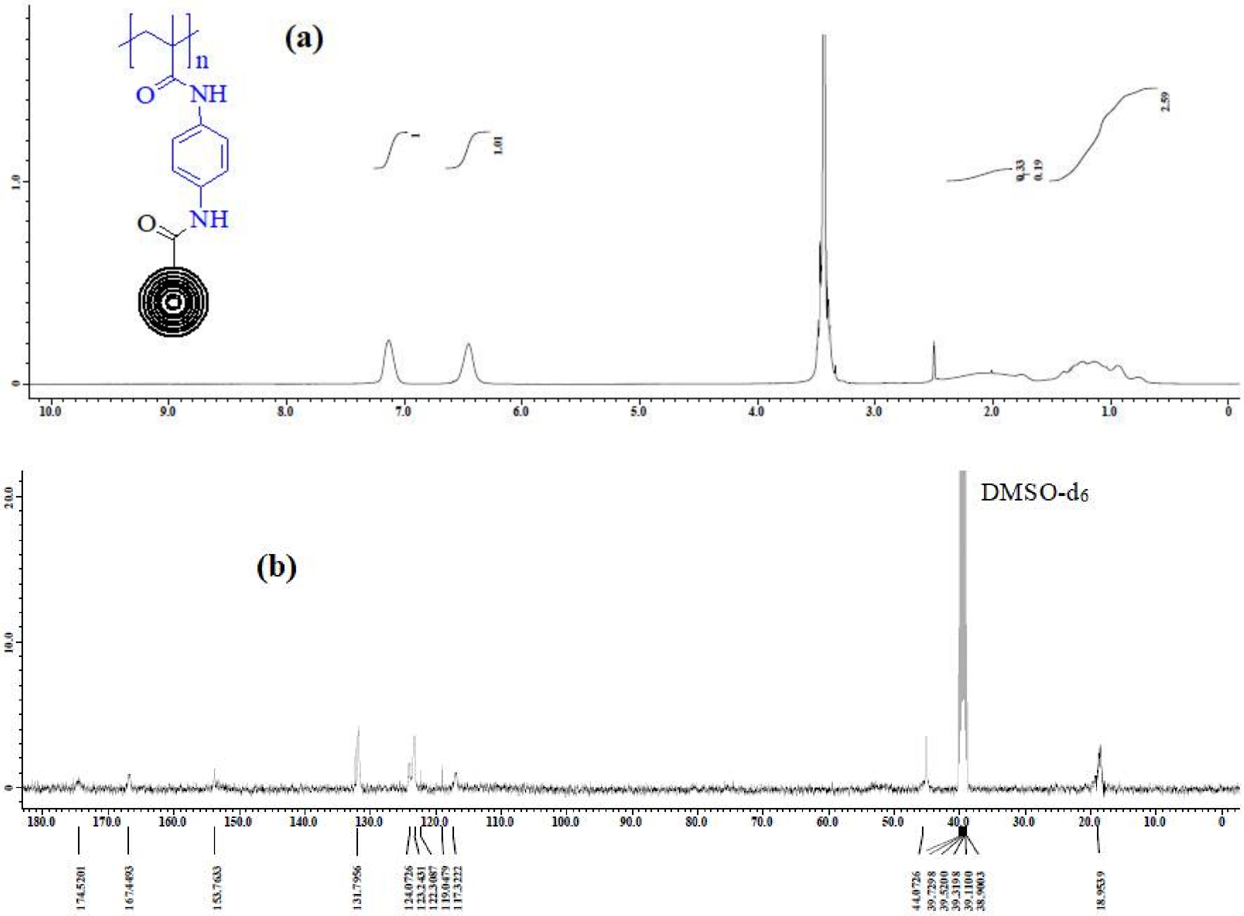

Figure S3. (a) ^{1}H-NMR, (b) ^{13}C-NMR spectra of PAPMA-CNOs.

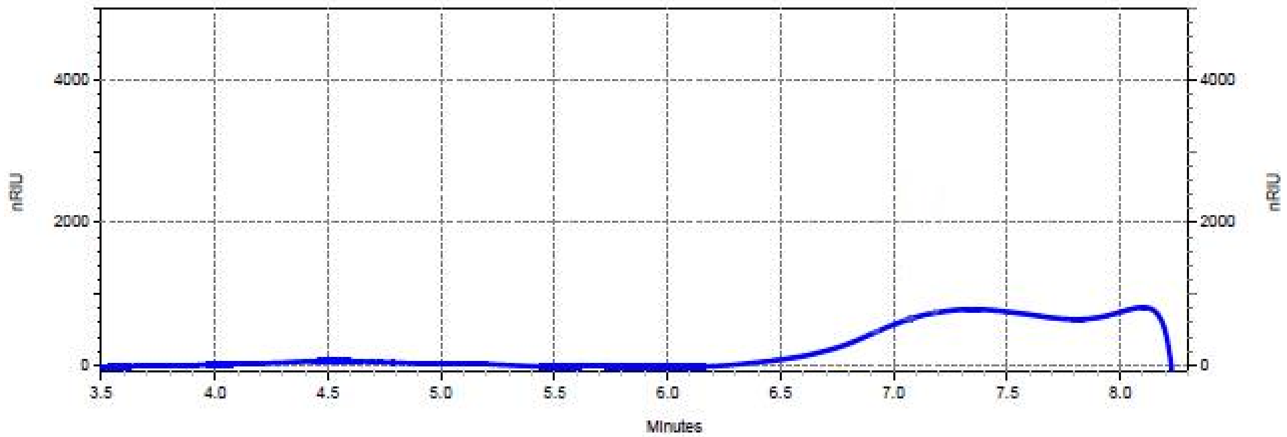

Figure S4. Gel permeation chromatography (GPC) curve of PAPMA.

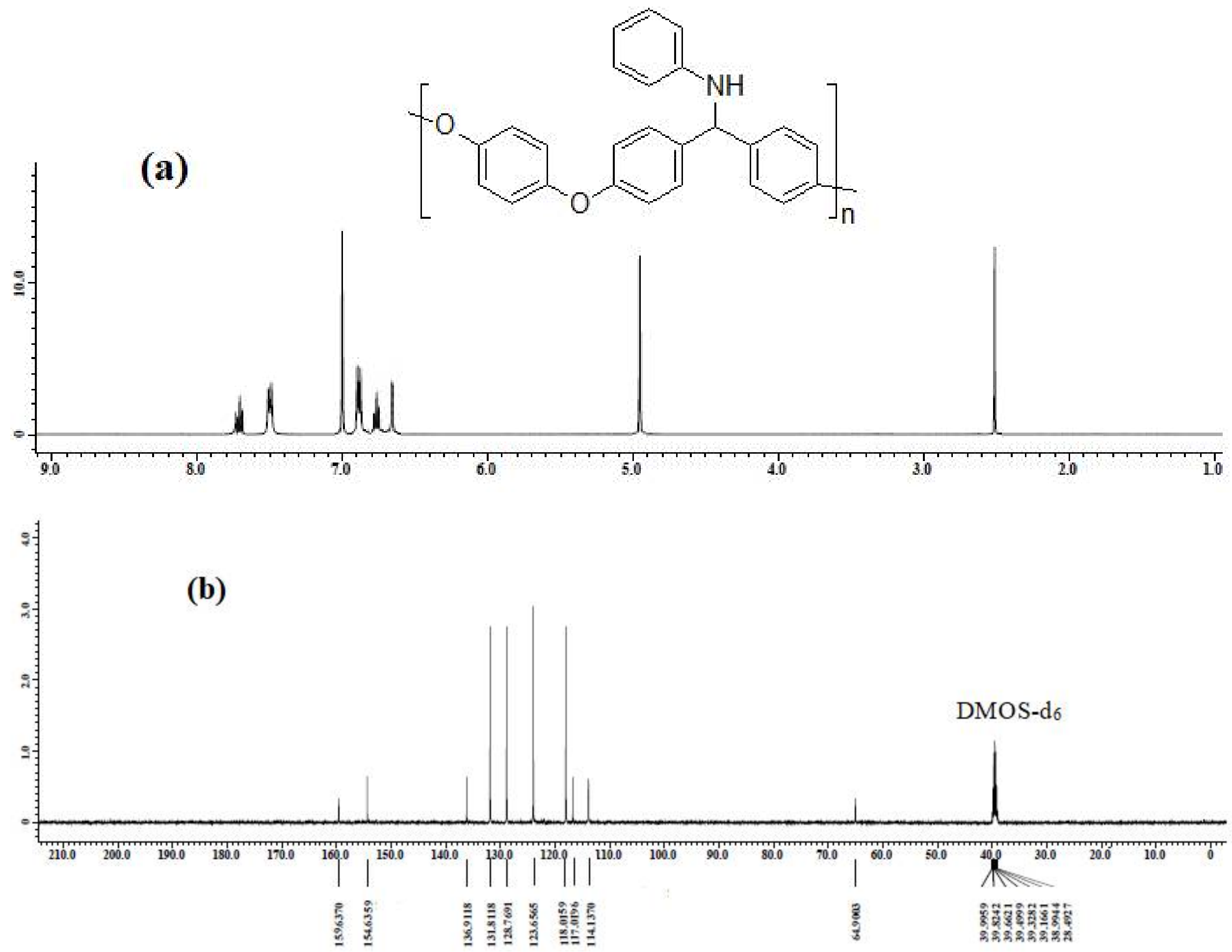

Figure S5. (a) ^{1}H-NMR, (b) ^{13}C-NMR spectra of AN-PEEK.

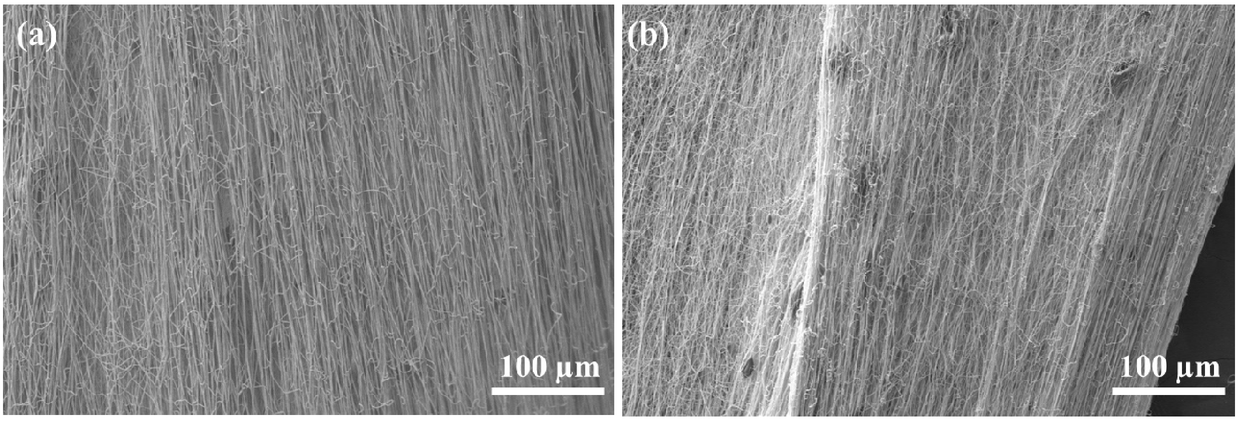

Figure S6. SEM micrographs of (a) AN-PEEK and (b) f-CNOs nanofibers.
